# Influenza Vaccination Coverage among Multiple Sclerosis Patients: Evolution over Time and Associated Factors

**DOI:** 10.3390/vaccines10071154

**Published:** 2022-07-21

**Authors:** Ignacio Hernández-García, Moisés Garcés-Redondo, Joana Rodríguez-Montolio, Irantzu Bengoa-Urrengoechea, Judit Espinosa-Rueda, Carlos Aibar-Remón, Vaccine Working Group

**Affiliations:** 1 Department of Preventive Medicine, Lozano Blesa University Clinical Hospital of Zaragoza, Calle San Juan Bosco 15, 50009 Zaragoza, Spain; ibengoa@salud.aragon.es (I.B.-U.); caibar@salud.aragon.es (C.A.-R.); 2 Health Services Research Group of Aragon (GRISSA), Aragon Institute for Health Research (IISA), Calle San Juan Bosco 15, 50009 Zaragoza, Spain; 3 Department of Neurology, Lozano Blesa University Clinical Hospital of Zaragoza, Calle San Juan Bosco 15, 50009 Zaragoza, Spain; mgarcesr@salud.aragon.es (M.G.-R.); jrodriguezm@salud.aragon.es (J.R.-M.); jespinosaru@salud.aragon.es (J.E.-R.); 4 Department of Preventive Medicine and Public Health, University of Zaragoza, Calle de Pedro Cerbuna 12, 50009 Zaragoza, Spain

**Keywords:** influenza vaccines, multiple sclerosis, Spain, vaccination coverage, associated factors

## Abstract

Our objective was to determine the influenza vaccination rate in a Spanish cohort of multiple sclerosis (MS) patients. A retrospective cohort study was carried out. Patients who attended the MS unit of the Lozano Blesa Hospital of Zaragoza between January 2015 and 2020 were included. The variables were obtained by reviewing the specialized and primary care records. Associations between receiving the vaccine in each flu season and the other variables were analyzed using bivariate analysis and multiple logistic regression models. A total of 260 patients were studied, with a median age of 31 years at the time of diagnosis. A total of 62.3% (162/260) were women. Vaccination coverage ranged from 20.4% in the 2015–2016 and 2016–2017 seasons to 41.5% in the 2019–2020 season (*p* = 0.000). Having been vaccinated in the previous season (ORa: 16.47–390.22; *p* = 0.000) and receiving a vaccination recommendation from the hospital vaccination unit (ORa: 2.44–3.96; *p* < 0.009) were associated with being vaccinated. The coverage is in an intermediate position compared to other countries. It is necessary to improve the referral system of these patients to the hospital vaccination unit because the information obtained by this service contributed to higher vaccination rates.

## 1. Introduction

Multiple sclerosis (MS) is an inflammatory demyelinating disease of the central nervous system and the leading cause of nontraumatic disability in young adults [1]. In particular, MS patients are at high risk of severe complications by some infectious diseases, such as influenza [2].

Annual administration of the influenza vaccine to people with MS is an internationally and nationally recommended measure [2,3,4,5] because it is the main method of preventing flu and its severe complications [2]. The influenza vaccine is effective and safe in MS patients [6]. It can limit the deleterious effects derived from fully manifesting flu; such infection can trigger glial activation, increased T cells, and neutrophil cerebral trafficking, as well as contribute towards more severe MS exacerbations [7]. However, influenza vaccination coverage in these patients in various countries, such as Germany [8], Italy [9], the USA [10], or Latin American countries [11], is lower than desirable, ranging from 19.0 [8] to 59.1% [10].

In Spain, the flu vaccine is administered free-of-charge in primary care centers to the groups of people recommended for vaccination by the Ministry of Health, and influenza vaccination coverage is an indicator of the quality of care, in terms of clinical effectiveness [12]. Since the 2014–2015 season, the Spanish Ministry of Health has recommended annual flu vaccination in people with chronic neurological diseases, including MS [13,14]. Nevertheless, specific data on the frequency of influenza vaccination in people with MS, or chronic neurological diseases in general, have not yet been published in Spain. It contrasts with what happens in other groups of chronic patients, for whom the Ministry of Health also recommends vaccination, in which several studies have described flu vaccination rates of 34.3% in diabetics [15], 36.3% in people with chronic respiratory disease [16], or 52.5% in people with chronic cardiovascular disease [17].

The aim of this study is to determine the changes in influenza vaccination coverage over time, as well as its associated factors, in a cohort of MS patients in a Spanish region.

## 2. Materials and Methods

A retrospective cohort study was carried out in Aragon (Spain); approximately 1,200,000 people receive health care in this Spanish region. The Lozano Blesa University Clinical Hospital (LBUCH) of Zaragoza has one of the two MS units in Aragon, in which MS patients are monitored at least every 6 months. Likewise, in preventive medicine service, there is a vaccination unit for risk groups, in which MS patients have been included since mid-2017. In this vaccination unit, among others, their vaccination status against pneumococcus, measles, and chickenpox is assessed and/or updated; they are also recommended to be vaccinated annually against influenza at their primary care center by means of brief advice (Table 1) [18].

### 2.1. Patients and Inclusion Criteria

Patients seen in the MS unit of the LBUCH between 1 January 2015 and 1 January 2020 were included. The inclusion criterion was having been diagnosed with MS before 3 October 2014 (the date on which flu vaccination public campaigns in Spain began to include MS patients as a target group for vaccination) [13].

### 2.2. Variables and Data Collection

Physicians of the MS and vaccination units of the LBUCH obtained the following information by reviewing the electronic medical records of primary care and specialized care: sex, date and country of birth, town of residence, allergies, date and age at MS diagnosis, type of MS (i.e, (a) relapsing-remitting (RR), (b) secondary progressive (SP), (c) clinically isolated syndrome (CIS), and d) primary progressive (PP) [19]), belonging to any other influenza vaccination target group (according to the recommendations of the Spanish Ministry of Health (Table 2) [13,14]), having received the flu vaccine in the previous season, having received information and medical advice at the vaccination unit of the LBUCH about the importance of being vaccinated annually against influenza at their primary care center, and date and place of administration of the influenza vaccine in the 2015–2016, 2016–2017, 2017–2018, 2018–2019, and 2019–2020 seasons.

### 2.3. Statistical Analysis

A descriptive analysis of all variables was performed. The town of residence variable was categorized into urban and rural. Likewise, bivariate analyses were performed, in which the dependent variable was considered to be having received the influenza vaccine in each of the flu seasons; the other variables were considered to be independent variables. The chi-square test or Fischer’s exact test were used for this purpose. In addition, multiple logistic regression analyses were performed with the variables for which a significant association was observed in the bivariate analyses. To quantify the associations, the adjusted odds ratios (aOR) were calculated with 95% confidence intervals (95% CI). Finally, influenza vaccination coverage was compared, according to the flu season, using McNemar’s test.

The level of statistical significance considered was *p* < 0.05, and the analysis program used was SPSS v.24.0. This study was approved by the Research Ethics Committee of Aragon (protocol code: C.P.–C.I. PI21/417).

## 3. Results

The number of patients under study was 260. A total of 62.3% (162/260) were women, with a median age of 31 years (range: 10–62 years) at the time of diagnosis. In total, 96.9% were born in Spain. According to the type of MS, 73.5% presented the RR type and 20.8% presented the SP type. No patient presented allergies to the components of the influenza vaccines (Table 3). There were five deaths during the study period (none caused by influenza or its vaccine).

Vaccination coverage ranged from 20.4% in the 2015–2016 and 2016–2017 seasons to 41.5% in the 2019–2020 season (*p* = 0.000) (Table 3 and Table 4).

In the bivariate analyses, the variables that were significantly associated with vaccine administration were: (a) influenza vaccination in the previous season; (b) age 65 or over, (c) town of residence, and (d) having received information and advice about being vaccinated from a preventive medicine service physician (Table 5). In the multivariate analyses, the variable that maintained a statistically significant association throughout all the seasons was the history of influenza vaccination in the previous season (aOR: 16.47–390.22; *p* = 0.000); for its part, receiving information and advice about being vaccinated from a preventive medicine service physician also maintained its statistically significant association, except in the 2020–2021 season (aOR: 2.44–3.96; *p* < 0.009) (Table 5).

## 4. Discussion

This study is the first to evaluate, in Spain, influenza vaccination coverage in MS patients. The observed vaccination rates (20.4–41.5%) are in an intermediate position, compared to those described internationally by other authors [8,9,10,11,20], which ranged from 19.0% in Germany [8] to 59.1% in the USA [10] in MS patients. However, the validity of this comparison could be limited by the fact that, unlike our study, such authors evaluated vaccination coverage in a single season.

On the other hand, these coverages are lower than those documented in Spain in other groups of patients targeted for influenza vaccination. Thus, in people with chronic respiratory diseases, Huedo et al. [16] described vaccination coverages of 36.3% and 35.0% in the 2013–2014 and 2016–2017 seasons, respectively, while Astray et al. [17] reported coverages of 37.4% and 36.3% in the 2011–2012 and 2013–2014 seasons, respectively. For their part, in diabetics, Huedo et al. [16] described coverages of 51.8% and 51.5% in the 2013–2014 and 2016–2017 seasons, respectively, while Astray et al. [17] reported coverages of 50.5% and 51.8% in the 2011–2012 and 2013–2014 seasons, respectively. Similarly, in people with chronic cardiovascular disease, Huedo et al. [16] described coverages of 51.1% and 51.3% in the 2013–2014 and 2016–2017 seasons, respectively, whereas Astray et al. [17] reported coverages of 52.5% and 51.1% in the 2011–2012 and 2013–2014 seasons, respectively.

No patient was allergic to the vaccine, and more than half had some other indication for vaccination (mainly by receiving immunosuppressive treatment) [14]. However, the percentage of unvaccinated patients was over 58%. These results could reflect the patients’ lack of awareness of the importance of receiving this vaccine or possible feelings of vaccine hesitancy. Each year’s vaccine varies; so, possible feelings of vaccine hesitancy may change, depending on information emerging with vaccination campaigns. As with any autoimmune condition, patients may be concerned about the risks related to vaccination, the humoral immune response, and flares or relapse of the underlying condition. All these aspects should be evaluated in a subsequent investigation. In any case, it is necessary to implement some improvement interventions, such as systematizing the referral of these patients to the hospital’s vaccination unit, given that, as observed in several seasons, the information and medical advice provided in this unit contributed to obtaining higher vaccination coverage. Likewise, this should be complementary to the recommendation of vaccination from primary care, where it has been documented that, in other groups of patients targeted for vaccination, advice to be vaccinated against influenza is an effective method to increase the vaccination rate [21].

In addition, improving patient access to the influenza vaccine by offering to administer it when they go to the hospital vaccination unit to receive the 13-valent pneumococcal conjugate or 23-valent polysaccharide vaccines could be another potential improvement measure to implement and evaluate. Enabling factors have been described as useful in improving influenza vaccination coverage in other target groups [22]. Moreover, pneumococcal and influenza vaccines can be administered in the same visit, without causing a decrease in the immune response or increase in the occurrence of adverse reactions [23].

The association observed, in all the seasons, between being vaccinated and having received the influenza vaccine in the previous season is congruent with that published in studies carried out in healthcare workers in Canada [24] and Mexico [25], as well as in other types of people subject to vaccination in Spain [26,27], in which previous vaccination has been described as an important predictor of vaccination. Similarly, the higher vaccination coverage observed in persons aged 65 years and older coincides with what other authors have described, since the influenza vaccine acceptance increases with age [28].

Unlike other countries, such as the USA or Canada, where the influenza vaccine is recommended to the general population from 6 months of age, in Spain, the vaccine is only recommended in certain population groups [13,14]. This did not allow us to use a general population control group to analyze whether having MS is a variable associated with influenza vaccination, an aspect that has been evaluated for MS and other diseases in studies conducted in the USA and Canada. Thus, in the USA, in the 2017–2018 season, Hung described significantly higher coverage among persons with diabetes (64.8%), compared with those without diabetes (43.9%) [29]; likewise, in the 2015–2016 season, Ruth described significantly higher coverage among persons with inflammatory bowel disease, compared with those without such disease (33.1% versus 23.6%), as well as in persons with MS (31.3% versus 24.1%) and rheumatoid arthritis (30.9% versus 23.1%) [30].

Among the limitations of our study is the sample size (260), which, despite being larger than that used by other authors (e.g., 101 [20] or 194 [9,31]), may have obtained results that were not very precise and had wide confidence intervals. Nevertheless, the mentioned sample size exceeded 236 persons, which was the minimum number of patients to be included, considering a precision of 5%, alpha error of 5%, and expected vaccination coverage of 19.0% [8]. Although our study was carried out in a single Spanish region, it provides a systematic evaluation of influenza vaccination coverage in these patients that could be implemented in the rest of Spain, a relevant fact, given that, up until now, there are only national data for three groups of persons targeted for vaccination (pregnant women, persons aged 65 years or older, and healthcare workers) [32]. Despite the fact that this was a registry study, there were no missing data, since all the data under study were recorded in the medical record. However, in this source of information was not collected, and, therefore, did not allow for the study of other variables potentially associated with vaccination, such as the socioeconomic status. Nevertheless, in countries such as ours, where the influenza vaccine is provided free-of-charge for patient groups included in public vaccination campaigns, it has recently been shown that influenza vaccination coverage does not differ by socioeconomic status [33].

## 5. Conclusions

There is scope for improvement in influenza vaccination coverage among MS patients. Knowing the factors associated with vaccination allows for proposing specific strategies to increase coverage, thus prioritizing subgroups in which to apply them, such as those under 65 years of age. The integration of different strategies will be essential, in order to increase influenza vaccine coverage [34,35,36]. Thus, all healthcare workers should recommend seasonal influenza vaccination to MS people; communication campaigns regarding the benefits of influenza vaccination should be undertaken and specifically targeted to these patients by age group. Public funding of flu vaccine for MS patients should be maintained, and the addition of pharmacists as providers of the influenza vaccine should be considered by public health authorities to further facilitate access to the vaccine.

## Figures and Tables

**Table 1:** Information and medical advice provided about flu vaccination.

Multiple sclerosis people are at high risk for complications by influenza.
Since the virus changes from year-to-year, you should be vaccinated every year at your primary care center.
Vaccination campaign usually starts in mid-October, after the national holiday (12 October).
Influenza vaccine administered at primary care centers is very safe. It is made up of killed viruses, so it is impossible for it to give you the flu.
The vaccine helps prevent you from getting the flu. In addition, if you are vaccinated but still get the flu, your illness is likely to be much milder than if you had not been vaccinated.

**Table 2:** Other target groups in which the Spanish Ministry of Health recommends influenza vaccination [13,14].

*Persons 65 years or older*
*Persons less than 65 years who are at high risk of complications by influenza*
Minors (aged 6 months and older) and adults with chronic cardiovascular disease (excluding isolated arterial hypertension) or chronic respiratory disease.
Minors (aged 6 months and older) and adults with:
Diabetes mellitus;
Morbid obesity;
Chronic kidney disease;
Hemoglobinopathies and anemias;
Asplenia;
Chronic liver disease;
Immunosuppression;
Cancer;
Cochlear implantation or awaiting cochlear implantation;
Disorders and diseases leading to cognitive dysfunction (Down Syndrome, dementia, or cognitive impairment);
Cerebrospinal fluid fistula ^1^;
Celiac disease ^1^;
Chronic inflammatory disease (inflammatory bowel disease-Crohn’s disease and ulcerative colitis-and inflammatory arthropathies-systemic erythematosus lupus, rheumatoid or juvenile arthritis-) ^1^.
*Pregnant women in any gestational trimester*

^1^ From the 2018–2019 season.

**Table 3:** Results of the descriptive analysis.

	N = 260
*Sex*, *n (%)*	
Woman	162 (62.3)
Man	98 (37.7)
*Median age at multiple sclerosis diagnosis (range)*, *years*	31 (10–62)
*Town of residence*, *n (%)*	
Urban	146 (56.2)
Rural	114 (43.8)
*Country of birth*, *n (%)*	
Spain	252 (96.9)
Bulgaria	2 (0.8)
Morocco	2 (0.8)
Others	4 (1.5)
*Multiple sclerosis type*, *n (%)*	
RR ^1^	191 (73.5)
SP ^2^	54 (20.8)
CIS ^3^	10 (3.8)
PP ^4^	5 (1.9)
*Influenza vaccination coverage*, *n (%)*	
2015–2016	53 (20.4)
2016–2017	53 (20.4)
2017–2018	80 (30.8)
2018–2019	107 (41.2)
2019–2020	108 (41.5)

Results expressed as absolute (*n*) and relative (%) frequencies. ^1^ RR: relapsing–remitting; ^2^ SP: secondary progressive; ^3^ CIS: clinically isolated syndrome: ^4^ PP: primary progressive.

**Table 4:** Comparison of vaccination coverage, according to season.

Vaccination Coverage	2015–2016	2016–2017	2017–2018	2018–2019
2015–2016	–			
2016–2017	*p* = 1.000	–		
2017–2018	*p* = 0.000	*p* = 0.000	–	
2018–2019	*p* = 0.000	*p* = 0.000	*p* = 0.000	–
2019–2020	*p* = 0.000	*p* = 0.000	*p* = 0.000	*p* = 1.000

**Table 5:** Results of the bivariate analysis and variables included in the logistic regression models.

	**Flu Vaccinated 2015–2016**		***p*** **^1^**	**aOR (95%CI)**	***p*** **^2^**
	**Yes (*n* = 53)**	**No (*n* = 207)**			
*Sex*, *n (%)*					
Man	22 (41.5)	76 (36.7)	0.520		
Woman	31 (58.5)	131 (63.3)			
*Vaccination previous season*, *n (%)*					
Yes	49 (92.5)	10 (4.8)	0.000	390.22 (83.12–1831.87)	0.000
No	4 (7.5)	197 (95.2)		1	
*Multiple sclerosis type*, *n (%)*					
RR ^3^	31 (58.5)	160 (77.3)	0.006		
CIS/PP ^4^	4 (7.5)	11 (5.3)	0.627		
SP ^5^	18 (34.0)	36 (17.4)			
*Country of birth*, *n (%)*					
Spain	53 (100)	199 (96.1)	0.366		
Others	0 (0)	8 (3.9)			
*Town of residence*, *n (%)*					
Urban	25 (47.2)	121 (58.5)	0.140		
Rural	28 (52.8)	86 (41.5)			
*Immunosuppression*, *n (%)*					
Yes	27 (50.9)	140 (67.6)	0.024		
No	26 (49.1)	67 (32.4)			
*Cognitive dysfunction*, *n (%)*					
Yes	2 (3.8)	0 (0)	0.041		
No	51 (96.2)	207 (100)			
*Chronic respiratory disease*, *n (%)*					
Yes	2 (3.8)	9 (4.3)	1.000		
No	51 (96.2)	198 (95.7)			
*Age*, *n (%)*					
65 years and older	12 (22.6)	6 (2.9)	0.000	34.67 (4.51–266.82)	0.001
Less than 65 years	41 (77.4)	201 (97.1)		1	
*Chronic kidney disease*, *n (%)*					
Yes	0 (0)	2 (1.0)	1.000		
No	53 (100)	205 (99.0)			
*Diabetes mellitus*, *n (%)*					
Yes	3 (5,7)	5 (2.4)	0.209		
No	50 (94.3)	202 (97.6)			
*Chronic cardiovascular disease*, *n (%)*					
Yes	3 (5.7)	0 (0)	0.008		
No	50 (94.3)	207 (100)			
	**Flu vaccinated 2016–2017**		***p*** **^1^**	**aOR (95%CI)**	***p*** **^2^**
	**Yes (*n* = 53)**	**No (*n* = 207)**			
*Sex*, *n (%)*					
Man	24 (45.3)	74 (35.7)	0.201		
Woman	29 (54.7)	133 (64.3)			
*Vaccination previous season*, *n (%)*					
Yes	41 (77.4)	12 (5.8)	0.000	55.52 (23.30–132.28)	0.000
No	12 (22.6)	195 (94.2)		1	
*Multiple sclerosis type*, *n (%)*					
RR ^3^	35 (66.0)	156 (75.4)	0.129		
CIS/PP ^4^	3 (5.7)	12 (5.8)	0.743		
SP ^5^	15 (28.3)	39 (18.8)			
*Country of birth*, *n (%)*					
Spain	52 (98.1)	200 (96.6)	1.000		
Others	1 (1.9)	7 (3.4)			
*Town of residence*, *n (%)*					
Urban	26 (49.1)	120 (58.0)	0.243		
Rural	27 (50.9)	87 (42.0)			
*Immunosuppression*, *n (%)*					
Yes	30 (56.6)	147 (71.0)	0.045		
No	23 (43.4)	60 (29.0)			
*Cognitive dysfunction*, *n (%)*					
Yes	1 (1.9)	1 (0.5)	0.367		
No	52 (98.1)	206 (99.5)			
*Chronic respiratory disease*, *n (%*)					
Yes	3 (5.7)	8 (3.9)	0.701		
No	50 (94.3)	199 (96.1)			
*Age*, *n (%)*			0.000		
65 years and older	13 (24.5)	6 (2.9)			
Less than 65 years	40 (75.5)	201 (97.1)			
*Chronic kidney disease*, *n (%)*					
Yes	1 (1.9)	2 (1.0)	0.497		
No	52 (98.1)	205 (99.0)			
*Diabetes mellitus*, *n (%)*					
Yes	3 (5.7)	5 (2.4)	0.209		
No	50 (94.3)	202 (97.6)			
*Chronic cardiovascular disease*, *n (%)*					
Yes	3 (5.7)	0 (0)	0.008		
No	50 (94.3)	207 (100)			
*Pregnancy*, *n (%)*					
Yes	1 (1.9)	1 (0.5)	0.367		
No	52 (98.1)	206 (99.5)			
	**Flu vaccinated 2017–2018**		***p*** **^1^**	**aOR (95%CI)**	***p*** **^2^**
	**Yes (*n* = 80)**	**No (*n* = 177)**			
*Sex*, *n (%)*					
Man	29 (36.3)	68 (38.4)	0.740		
Woman	51 (63.7)	109 (61.6)			
*Vaccination previous season*, *n (%)*					
Yes	44 (55.0)	8 (4.5)	0.000	27.60 (11.63–65.49)	0.000
No	36 (45.0)	169 (95.5)		1	
*Multiple sclerosis type*, *n (%)*					
RR ^3^	52 (65.0)	137 (77.4)	0.028		
CIS/PP ^4^	5 (6.2)	10 (5.7)	0.488		
SP ^5^	23 (28.8)	30 (16.9)			
*Vaccination Unit advice*, *n (%)*					
Yes	15 (18.8)	11 (6.2)	0.002	3.96 (1.47–10.64)	0.006
No	65 (81.2)	166 (93.8)		1	
*Country of birth*, *n (%)*					
Spain	79 (98.8)	170 (96.0)	0.441		
Others	1 (1.2)	7 (4.0)			
*Town of residence*, *n (%)*					
Rural	44 (55.0)	69 (39.0)	0.017	2.15 (1.09–4.22)	0.026
Urban	36 (45.0)	108 (61.0)		1	
*Immunosuppression*, *n (%)*					
Yes	53 (66.3)	128 (72.3)	0.324		
No	27 (33.7)	49 (27.7)			
*Cognitive dysfunction*, *n (%)*					
Yes	1 (1.3)	1 (0.6)	0.527		
No	79 (98.7)	176 (99.4)			
*Chronic respiratory disease*, *n (%)*					
Yes	2 (2.5)	9 (5.1)	0.511		
No	78 (97.5)	168 (94.9)			
*Age*, *n (%)*					
65 years and older	12 (15.0)	8 (4.5)	0.004		
Less than 65 years	68 (85.0)	169 (95.5)			
*Chronic kidney disease*, *n (%)*					
Yes	1 (1.3)	2 (1.1)	1.000		
No	79 (98.7)	175 (98.9)			
*Diabetes mellitus*, *n (%)*					
Yes	3 (3.8)	6 (3.4)	1.000		
No	77 (96.2)	171 (96.6)			
*Chronic cardiovascular disease*, *n (%)*					
Yes	3 (3.8)	0 (0)	0.029		
No	77 (96.2)	177 (100)			
*Pregnancy*, *n (%)*					
Yes	1 (1.3)	1 (0.6)	0.527		
No	79 (98.7)	176 (99.4)			
	**Flu vaccinated 2018–2019**		***p*** **^1^**	**aOR (95%CI)**	***p*** **^2^**
	**Yes (*n* = 107)**	**No (*n* = 149)**			
*Sex*, *n (%)*					
Man	40 (37.4)	56 (37.6)	0.974		
Woman	67 (62.6)	93 (62.4)			
*Vaccination previous season*, *n (%)*					
Yes	70 (65.4)	10 (6.7)	0.000	27.39 (12.55–59.79)	0.000
No	37 (34.6)	139 (93.3)		1	
*Multiple sclerosis type*, *n (%)*					
RR ^3^	74 (69.2)	114 (76.5)	0.207		
CIS/PP ^4^	7 (6.5)	8 (5.4)	0.871		
SP ^5^	26 (24.3)	27 (18.1)			
*Vaccination Unit advice*, *n (%)*					
Yes	53 (49.5)	38 (25.5)	0.000	3.15 (1.60–6.18)	0.001
No	54 (50.5)	111 (74.5)		1	
*Country of birth*, *n (%)*					
Spain	105 (98.1)	143 (96.0)	0.475		
Others	2 (1.9)	6 (4.0)			
*Town of residence*, *n (%)*					
Urban	50 (46.7)	93 (62.4)	0.013		
Rural	57 (53.3)	56 (37.6)			
*Immunosuppression*, *n (%)*					
Yes	75 (70.1)	110 (73.8)	0.511		
No	32 (29.9)	39 (26.2)			
*Cognitive dysfunction*, *n (%)*					
Yes	2 (1.9)	0 (0)	0.174		
No	105 (98.1)	149 (100)			
*Chronic respiratory disease*, *n (%)*					
Yes	3 (2.8)	8 (5.4)	0.368		
No	104 (97.2)	141 (94.6)			
*Age*, *n (%)*					
65 years and older	14 (13.1)	7 (4.7)	0.016		
Less than 65 years	93 (86.9)	142 (95.3)			
*Chronic kidney disease*, *n (%)*					
Yes	2 (1.9)	1 (0.7)	0.573		
No	105 (98.1)	148 (99.3)			
*Diabetes mellitus*, *n (%)*					
Yes	3 (2.8)	6 (4.0)	0.739		
No	104 (97.2)	143 (96.0)			
*Chronic cardiovascular disease*, *n (%)*					
Yes	5 (4.7)	0 (0)	0.012		
No	102 (95.3)	149 (100)			
*Pregnancy*, *n (%)*					
Yes	0 (0)	2 (1.3)	0.512		
No	107 (100)	147 (98.7)			
*Celiac disease*, *n (%)*					
Yes	1 (0.9)	1 (0.7)	1.000		
No	106 (99.1)	148 (99.3)			
*Chronic inflammatory disease*, *n (%)*^6^					
Yes	1 (0.9)	2 (1.3)	1.000		
No	106 (99.1)	147 (98.7)			
	**Flu vaccinated 2019–2020**		***p*** **^1^**	**aOR (95%CI)**	***p*** **^2^**
	**Yes (*n* = 108)**	**No (*n* = 148)**			
*Sex*, *n (%)*					
Man	38 (35.2)	58 (39.2)	0.513		
Woman	70 (64.8)	90 (60.8)			
*Vaccination previous season*, *n (%)*					
Yes	84 (77.8)	23 (15.5)	0.000	16.47 (8.61–31.50)	0.000
No	24 (22.2)	125 (84.5)		1	
*Multiple sclerosis type*, *n (%)*					
RR ^3^	74 (68.5)	114 (77.0)	0.080		
CIS/PP ^4^	6 (5.6)	9 (6.1)	0.384		
SP ^5^	28 (25.9)	25 (16.9)			
*Vaccination Unit advice*, *n (%)*					
Yes	58 (53.7)	45 (30.4)	0.000	2.44 (1.25–4.77)	0.009
No	50 (46.3)	103 (69.6)		1	
*Country of birth*, *n (%)*					
Spain	107 (99.1)	141 (95.3)	0.144		
Others	1 (0.9)	7 (4.7)			
*Town of residence*, *n (%)*					
Urban	53 (49.1)	90 (60.8)	0.062		
Rural	55 (50.9)	58 (39.2)			
*Immunosuppression*, *n (%)*					
Yes	72 (66.7)	114 (77.0)	0.066		
No	36 (33.3)	34 (23.0)			
*Cognitive dysfunction*, *n (%)*					
Yes	2 (1.9)	0 (0)	0.177		
No	106 (98.1)	148 (100)			
*Chronic respiratory disease*, *n (%)*					
Yes	3 (2.8)	9 (6.1)	0.217		
No	105 (97.2)	139 (93.9)			
*Age*, *n (%)*					
65 years and older	15 (13.9)	6 (4.1)	0.005	4.07 (1.16–14.26)	0.028
Less than 65 years	93 (86.1)	142 (95.9)		1	
*Chronic kidney disease*, *n (%)*					
Yes	0 (0)	3 (2.0)	0.265		
No	108 (100)	145 (98.0)			
*Diabetes mellitus*, *n (%)*					
Yes	7 (6.5)	4 (2.7)	0.211		
No	101 (93.5)	144 (97.3)			
*Chronic cardiovascular disease*, *n (%)*					
Yes	5 (4.6)	0 (0)	0.013		
No	103 (95.4)	148 (100)			
*Celiac disease*, *n (%)*					
Yes	1 (0.9)	2 (1.4)	1.000		
No	107 (99.1)	146 (98.6)			
*Chronic inflammatory disease*, *n (%)*^6^					
Yes	2 (1.9)	1 (0.7)	0.575		
No	106 (98.1)	147 (99.3)			

aOR (95%CI): adjusted odds ratio (95% confidence interval). ^1^ Significance level in bivariate analysis. ^2^ Significance level in multivariate analysis. ^3^ RR: relapsing–remitting; ^4^ CIS/PP: clinically isolated syndrome/primary progressive; ^5^ SP: secondary progressive. ^6^ Inflammatory bowel disease or systemic lupus erythematosus.

## Data Availability

Data are contained within the article.
